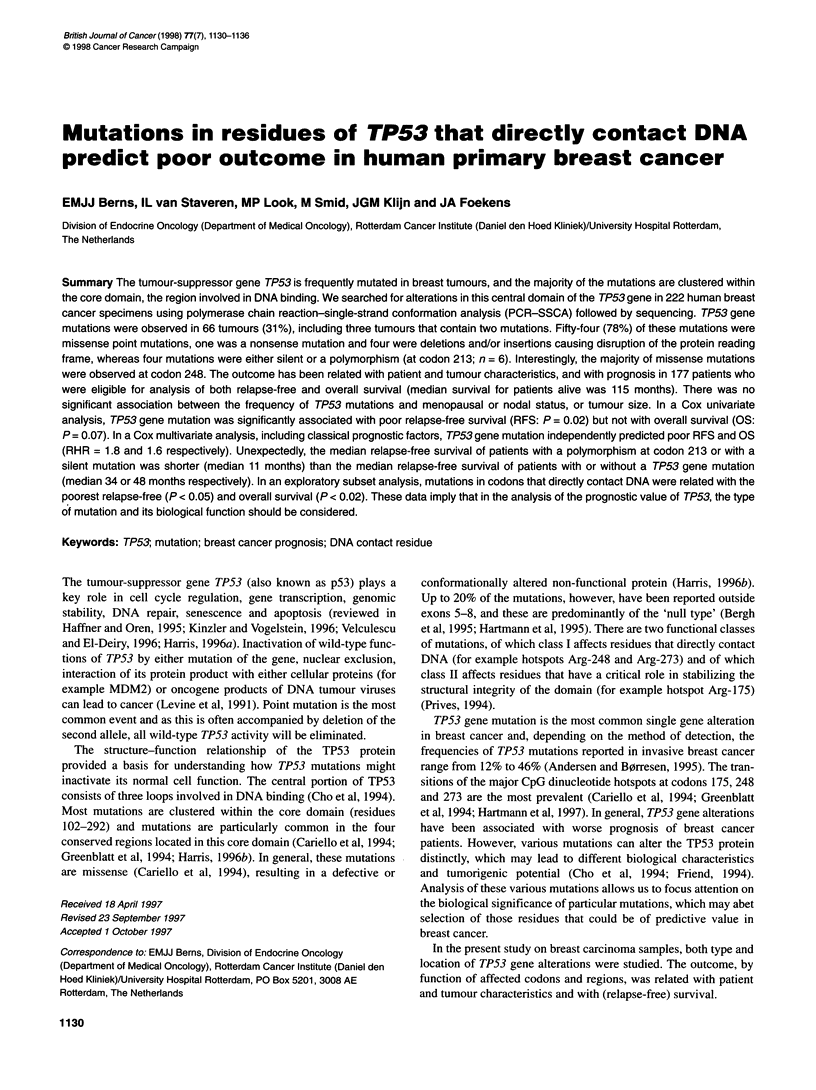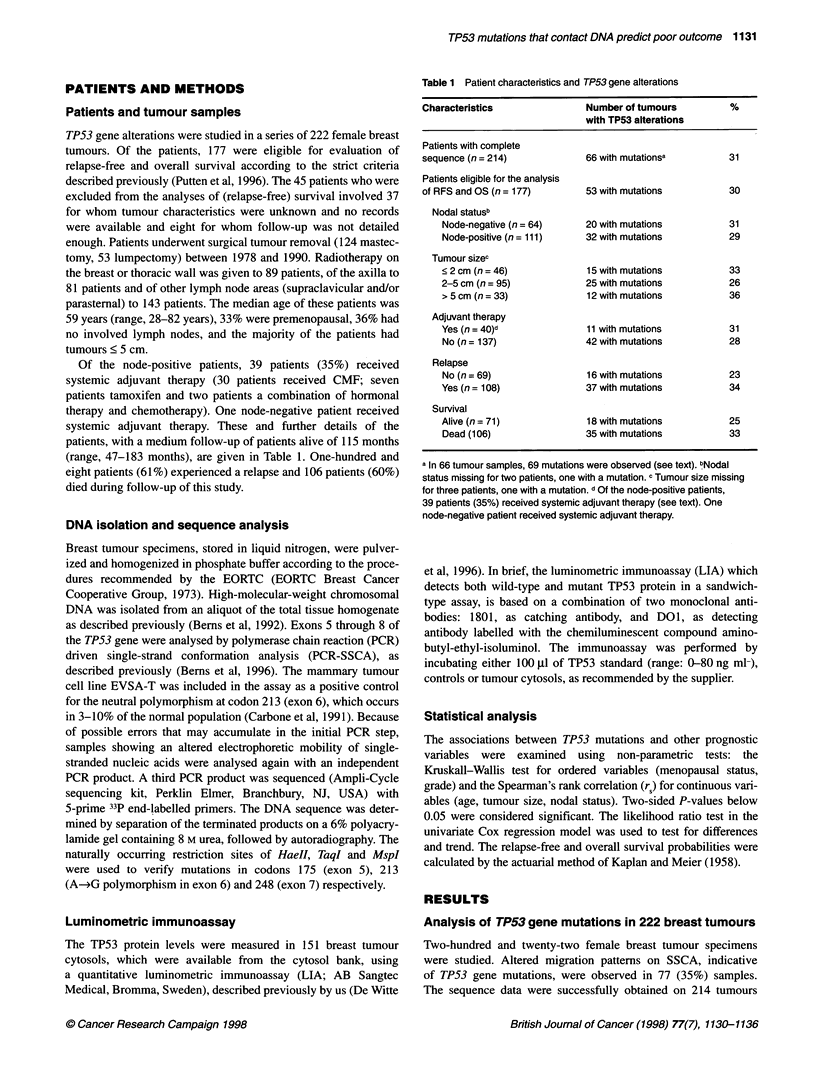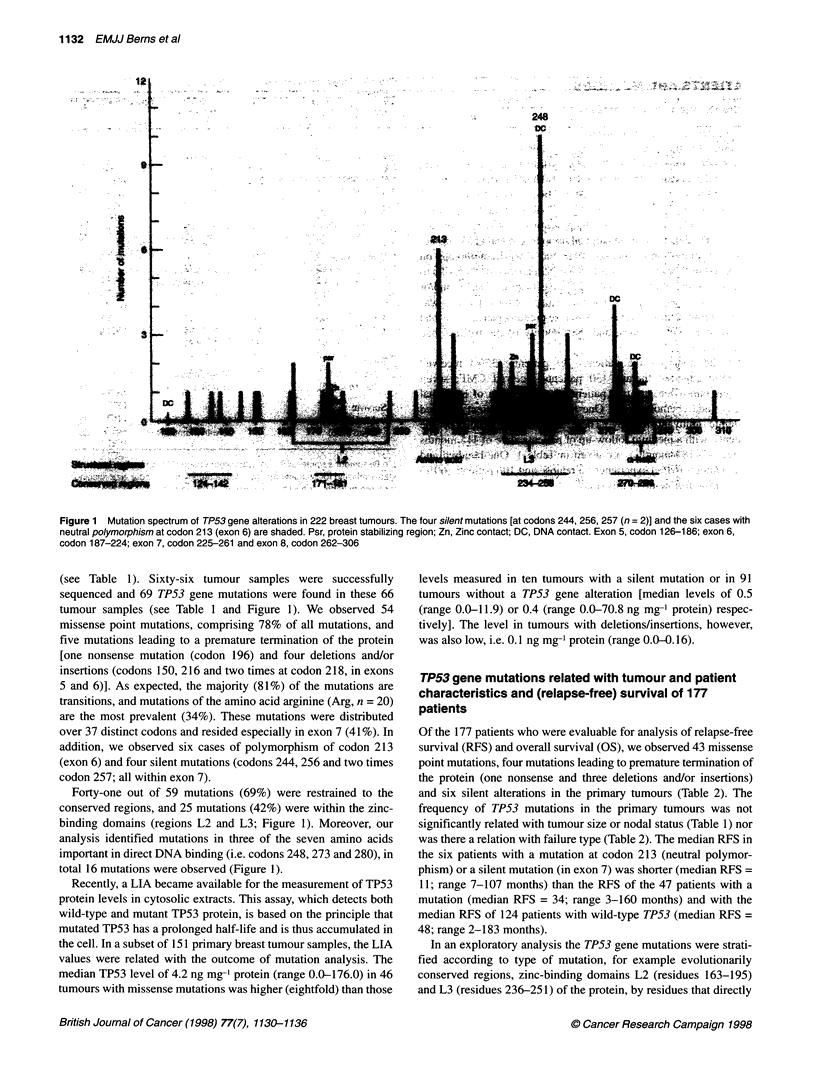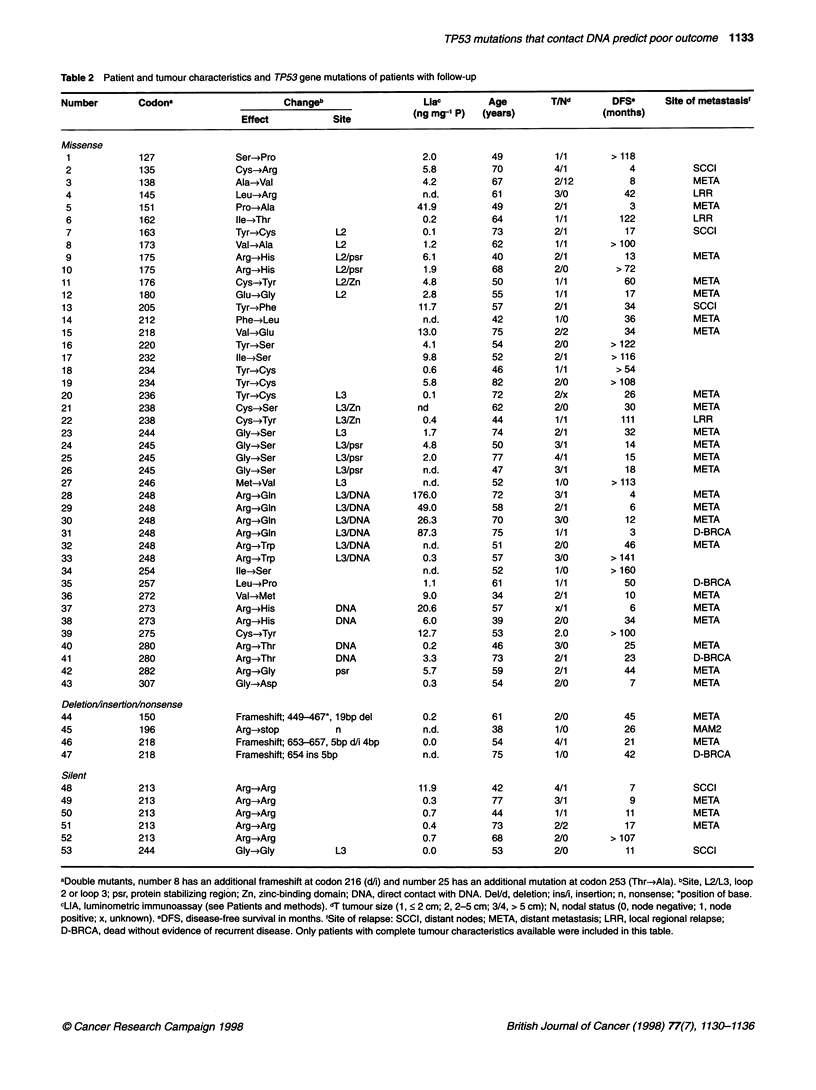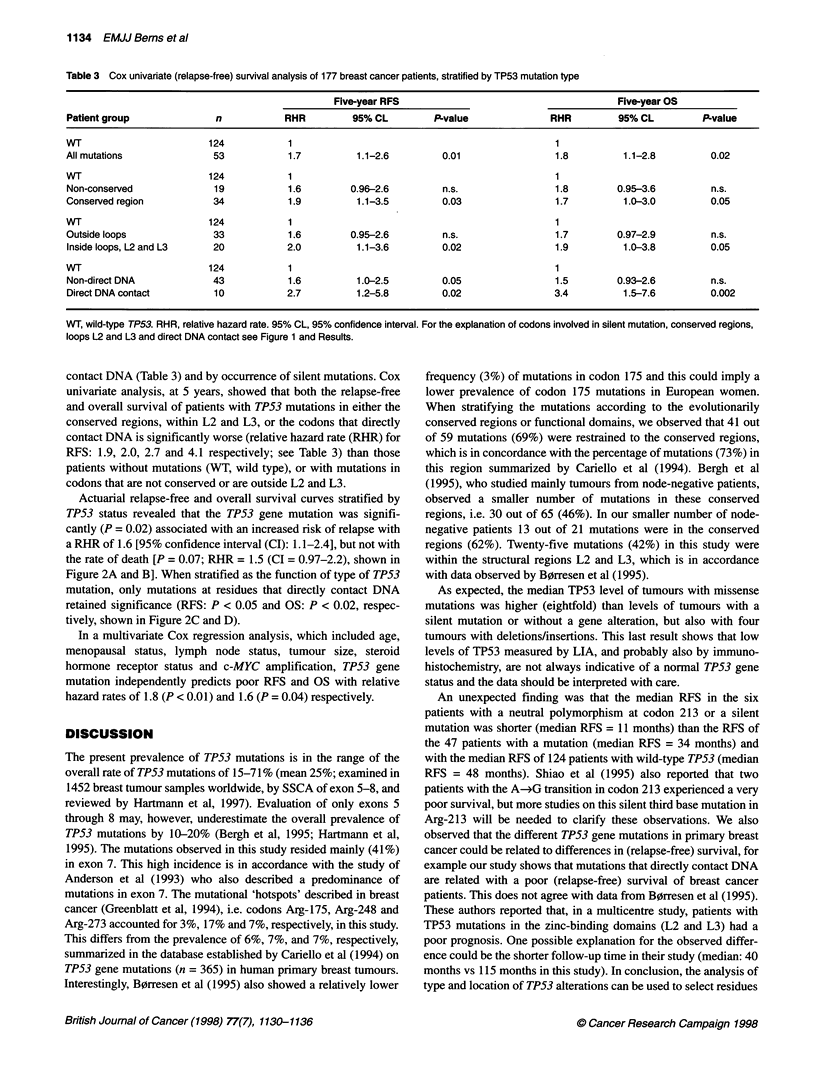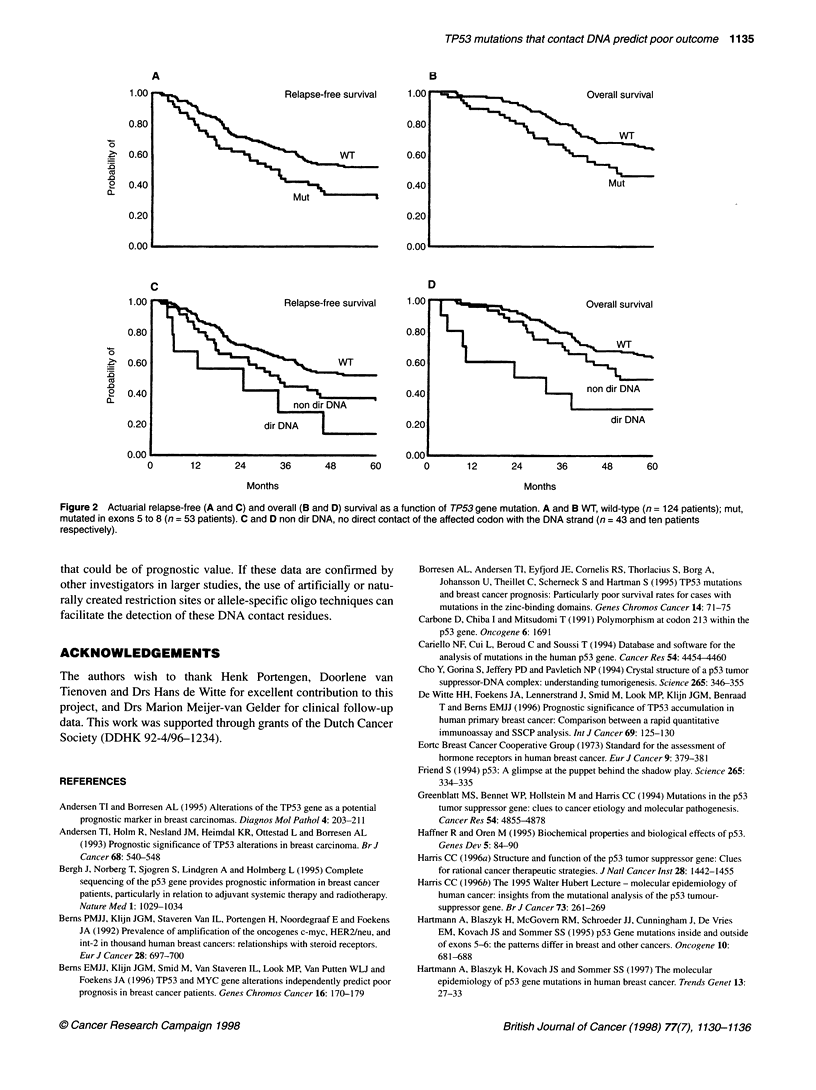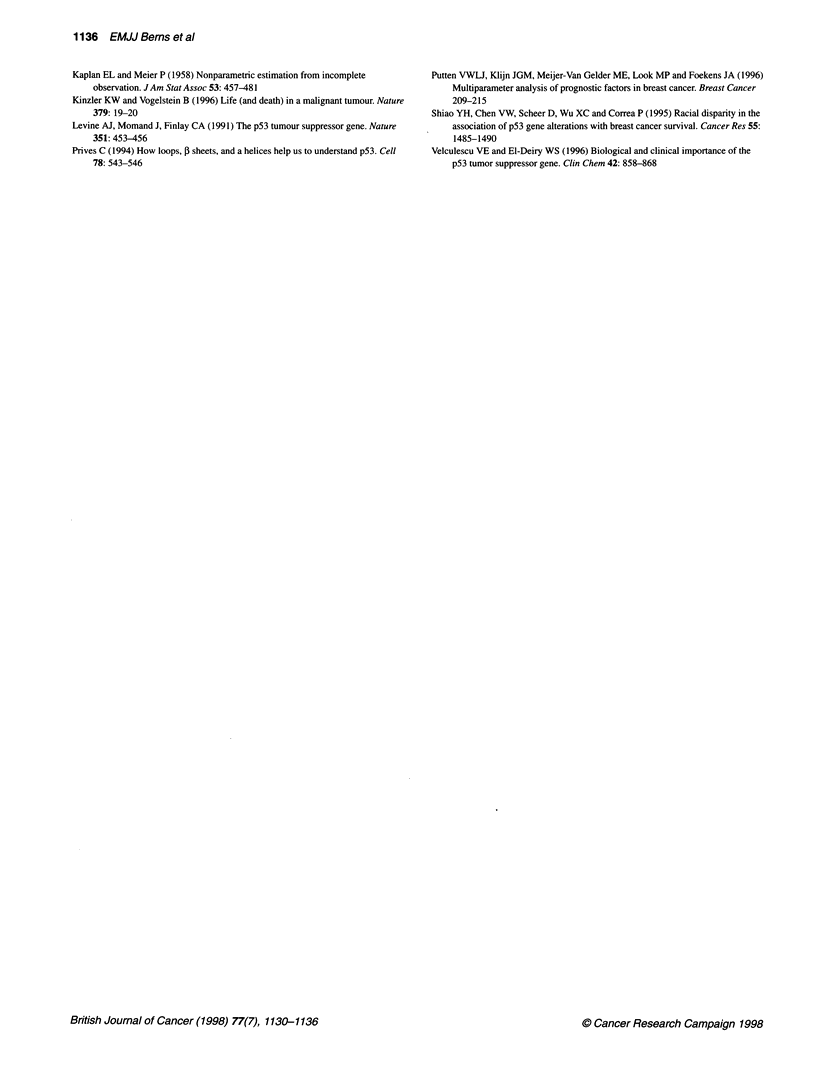# Mutations in residues of TP53 that directly contact DNA predict poor outcome in human primary breast cancer.

**DOI:** 10.1038/bjc.1998.187

**Published:** 1998-04

**Authors:** E. M. Berns, I. L. van Staveren, M. P. Look, M. Smid, J. G. Klijn, J. A. Foekens

**Affiliations:** Department of Medical Oncology, Rotterdam Cancer Institute (Daniel den Hoed Kliniek)/University Hospital Rotterdam, The Netherlands.

## Abstract

The tumour-suppressor gene TP53 is frequently mutated in breast tumours, and the majority of the mutations are clustered within the core domain, the region involved in DNA binding. We searched for alterations in this central domain of the TP53gene in 222 human breast cancer specimens using polymerase chain reaction-single-strand conformation analysis (PCR-SSCA) followed by sequencing. TP53 gene mutations were observed in 66 tumours (31%), including three tumours that contain two mutations. Fifty-four (78%) of these mutations were missense point mutations, one was a nonsense mutation and four were deletions and/or insertions causing disruption of the protein reading frame, whereas four mutations were either silent or a polymorphism (at codon 213; n = 6). Interestingly, the majority of missense mutations were observed at codon 248. The outcome has been related with patient and tumour characteristics, and with prognosis in 177 patients who were eligible for analysis of both relapse-free and overall survival (median survival for patients alive was 115 months). There was no significant association between the frequency of TP53 mutations and menopausal or nodal status, or tumour size. In a Cox univariate analysis, TP53 gene mutation was significantly associated with poor relapse-free survival (RFS: P = 0.02) but not with overall survival (OS: P = 0.07). In a Cox multivariate analysis, including classical prognostic factors, TP53 gene mutation independently predicted poor RFS and OS (RHR = 1.8 and 1.6 respectively). Unexpectedly, the median relapse-free survival of patients with a polymorphism at codon 213 or with a silent mutation was shorter (median 11 months) than the median relapse-free survival of patients with or without a TP53 gene mutation (median 34 or 48 months respectively). In an exploratory subset analysis, mutations in codons that directly contact DNA were related with the poorest relapse-free (P < 0.05) and overall survival (P < 0.02). These data imply that in the analysis of the prognostic value of TP53, the type of mutation and its biological function should be considered.


British Journal of Cancer (1998) 77(7), 1130–1136
© 1998 Cancer Research Campaign

### Mutations in residues of *TP53* that directly contact DNA predict poor outcome in human primary breast cancer

**EMJJ Berns, IL van Staveren, MP Look, M Smid, JGM Klijn and JA Foekens**

Division of Endocrine Oncology (Department of Medical Oncology), Rotterdam Cancer Institute (Daniel den Hoed Kliniek)/University Hospital Rotterdam, The Netherlands

**Summary** The tumour-suppressor gene *TP53* is frequently mutated in breast tumours, and the majority of the mutations are clustered within the core domain, the region involved in DNA binding. We searched for alterations in this central domain of the *TP53* gene in 222 human breast cancer specimens using polymerase chain reaction–single-strand conformation analysis (PCR–SSCA) followed by sequencing. *TP53* gene mutations were observed in 66 tumours (31%), including three tumours that contain two mutations. Fifty-four (78%) of these mutations were missense point mutations, one was a nonsense mutation and four were deletions and/or insertions causing disruption of the protein reading frame, whereas four mutations were either silent or a polymorphism (at codon 213; $n = 6$). Interestingly, the majority of missense mutations were observed at codon 248. The outcome has been related with patient and tumour characteristics, and with prognosis in 177 patients who were eligible for analysis of both relapse-free and overall survival (median survival for patients alive was 115 months). There was no significant association between the frequency of *TP53* mutations and menopausal or nodal status, or tumour size. In a Cox univariate analysis, *TP53* gene mutation was significantly associated with poor relapse-free survival (RFS: $P = 0.02$) but not with overall survival (OS: $P = 0.07$). In a Cox multivariate analysis, including classical prognostic factors, *TP53* gene mutation independently predicted poor RFS and OS (RHR = 1.8 and 1.6 respectively). Unexpectedly, the median relapse-free survival of patients with a polymorphism at codon 213 or with a silent mutation was shorter (median 11 months) than the median relapse-free survival of patients with or without a *TP53* gene mutation (median 34 or 48 months respectively). In an exploratory subset analysis, mutations in codons that directly contact DNA were related with the poorest relapse-free ($P < 0.05$) and overall survival ($P < 0.02$). These data imply that in the analysis of the prognostic value of *TP53*, the type of mutation and its biological function should be considered.

**Keywords:** *TP53*; mutation; breast cancer prognosis; DNA contact residue

The tumour-suppressor gene *TP53* (also known as p53) plays a key role in cell cycle regulation, gene transcription, genomic stability, DNA repair, senescence and apoptosis (reviewed in Haffner and Oren, 1995; Kinzler and Vogelstein, 1996; Velculescu and El-Deiry, 1996; Harris, 1996*a*). Inactivation of wild-type functions of *TP53* by either mutation of the gene, nuclear exclusion, interaction of its protein product with either cellular proteins (for example MDM2) or oncogene products of DNA tumour viruses can lead to cancer (Levine et al, 1991). Point mutation is the most common event and as this is often accompanied by deletion of the second allele, all wild-type *TP53* activity will be eliminated.

The structure–function relationship of the TP53 protein provided a basis for understanding how *TP53* mutations might inactivate its normal cell function. The central portion of TP53 consists of three loops involved in DNA binding (Cho et al, 1994). Most mutations are clustered within the core domain (residues 102–292) and mutations are particularly common in the four conserved regions located in this core domain (Cariello et al, 1994; Greenblatt et al, 1994; Harris, 1996*b*). In general, these mutations are missense (Cariello et al, 1994), resulting in a defective or conformationally altered non-functional protein (Harris, 1996*b*). Up to 20% of the mutations, however, have been reported outside exons 5–8, and these are predominantly of the 'null type' (Bergh et al, 1995; Hartmann et al, 1995). There are two functional classes of mutations, of which class I affects residues that directly contact DNA (for example hotspots Arg-248 and Arg-273) and of which class II affects residues that have a critical role in stabilizing the structural integrity of the domain (for example hotspot Arg-175) (Prives, 1994).

*TP53* gene mutation is the most common single gene alteration in breast cancer and, depending on the method of detection, the frequencies of *TP53* mutations reported in invasive breast cancer range from 12% to 46% (Andersen and Børresen, 1995). The transitions of the major CpG dinucleotide hotspots at codons 175, 248 and 273 are the most prevalent (Cariello et al, 1994; Greenblatt et al, 1994; Hartmann et al, 1997). In general, *TP53* gene alterations have been associated with worse prognosis of breast cancer patients. However, various mutations can alter the TP53 protein distinctly, which may lead to different biological characteristics and tumorigenic potential (Cho et al, 1994; Friend, 1994). Analysis of these various mutations allows us to focus attention on the biological significance of particular mutations, which may abet selection of those residues that could be of predictive value in breast cancer.

In the present study on breast carcinoma samples, both type and location of *TP53* gene alterations were studied. The outcome, by function of affected codons and regions, was related with patient and tumour characteristics and with (relapse-free) survival.

*Received 18 April 1997*
*Revised 23 September 1997*
*Accepted 1 October 1997*

*Correspondence to:* EMJJ Berns, Division of Endocrine Oncology (Department of Medical Oncology), Rotterdam Cancer Institute (Daniel den Hoed Kliniek)/University Hospital Rotterdam, PO Box 5201, 3008 AE Rotterdam, The Netherlands

### PATIENTS AND METHODS

### Patients and tumour samples

*TP53* gene alterations were studied in a series of 222 female breast tumours. Of the patients, 177 were eligible for evaluation of relapse-free and overall survival according to the strict criteria described previously (Putten et al, 1996). The 45 patients who were excluded from the analyses of (relapse-free) survival involved 37 for whom tumour characteristics were unknown and no records were available and eight for whom follow-up was not detailed enough. Patients underwent surgical tumour removal (124 mastectomy, 53 lumpectomy) between 1978 and 1990. Radiotherapy on the breast or thoracic wall was given to 89 patients, of the axilla to 81 patients and of other lymph node areas (supraclavicular and/or parasternal) to 143 patients. The median age of these patients was 59 years (range, 28–82 years), 33% were premenopausal, 36% had no involved lymph nodes, and the majority of the patients had tumours ≤ 5 cm.

Of the node-positive patients, 39 patients (35%) received systemic adjuvant therapy (30 patients received CMF; seven patients tamoxifen and two patients a combination of hormonal therapy and chemotherapy). One node-negative patient received systemic adjuvant therapy. These and further details of the patients, with a medium follow-up of patients alive of 115 months (range, 47–183 months), are given in Table 1. One-hundred and eight patients (61%) experienced a relapse and 106 patients (60%) died during follow-up of this study.

**Table 1** Patient characteristics and *TP53* gene alterations

| Characteristics | Number of tumours with TP53 alterations | % |
|---|---|---|
| Patients with complete sequence ($n$ = 214) | 66 with mutations[a] | 31 |
| Patients eligible for the analysis of RFS and OS ($n$ = 177) | 53 with mutations | 30 |
| Nodal status[b] | | |
| Node-negative ($n$ = 64) | 20 with mutations | 31 |
| Node-positive ($n$ = 111) | 32 with mutations | 29 |
| Tumour size[c] | | |
| ≤ 2 cm ($n$ = 46) | 15 with mutations | 33 |
| 2–5 cm ($n$ = 95) | 25 with mutations | 26 |
| > 5 cm ($n$ = 33) | 12 with mutations | 36 |
| Adjuvant therapy | | |
| Yes ($n$ = 40)[d] | 11 with mutations | 31 |
| No ($n$ = 137) | 42 with mutations | 28 |
| Relapse | | |
| No ($n$ = 69) | 16 with mutations | 23 |
| Yes ($n$ = 108) | 37 with mutations | 34 |
| Survival | | |
| Alive ($n$ = 71) | 18 with mutations | 25 |
| Dead (106) | 35 with mutations | 33 |

[a] In 66 tumour samples, 69 mutations were observed (see text). [b]Nodal status missing for two patients, one with a mutation. [c] Tumour size missing for three patients, one with a mutation. [d] Of the node-positive patients, 39 patients (35%) received systemic adjuvant therapy (see text). One node-negative patient received systemic adjuvant therapy.

### DNA isolation and sequence analysis

Breast tumour specimens, stored in liquid nitrogen, were pulverized and homogenized in phosphate buffer according to the procedures recommended by the EORTC (EORTC Breast Cancer Cooperative Group, 1973). High-molecular-weight chromosomal DNA was isolated from an aliquot of the total tissue homogenate as described previously (Berns et al, 1992). Exons 5 through 8 of the *TP53* gene were analysed by polymerase chain reaction (PCR) driven single-strand conformation analysis (PCR-SSCA), as described previously (Berns et al, 1996). The mammary tumour cell line EVSA-T was included in the assay as a positive control for the neutral polymorphism at codon 213 (exon 6), which occurs in 3–10% of the normal population (Carbone et al, 1991). Because of possible errors that may accumulate in the initial PCR step, samples showing an altered electrophoretic mobility of single-stranded nucleic acids were analysed again with an independent PCR product. A third PCR product was sequenced (Ampli-Cycle sequencing kit, Perklin Elmer, Branchbury, NJ, USA) with 5-prime $^{33}$P end-labelled primers. The DNA sequence was determined by separation of the terminated products on a 6% polyacrylamide gel containing 8 M urea, followed by autoradiography. The naturally occurring restriction sites of *HaeII*, *TaqI* and *MspI* were used to verify mutations in codons 175 (exon 5), 213 (A→G polymorphism in exon 6) and 248 (exon 7) respectively.

### Luminometric immunoassay

The TP53 protein levels were measured in 151 breast tumour cytosols, which were available from the cytosol bank, using a quantitative luminometric immunoassay (LIA; AB Sangtec Medical, Bromma, Sweden), described previously by us (De Witte et al, 1996). In brief, the luminometric immunoassay (LIA) which detects both wild-type and mutant TP53 protein in a sandwich-type assay, is based on a combination of two monoclonal antibodies: 1801, as catching antibody, and DO1, as detecting antibody labelled with the chemiluminescent compound amino-butyl-ethyl-isoluminol. The immunoassay was performed by incubating either 100 µl of TP53 standard (range: 0–80 ng ml$^{-}$), controls or tumour cytosols, as recommended by the supplier.

### Statistical analysis

The associations between *TP53* mutations and other prognostic variables were examined using non-parametric tests: the Kruskall–Wallis test for ordered variables (menopausal status, grade) and the Spearman's rank correlation ($r_s$) for continuous variables (age, tumour size, nodal status). Two-sided *P*-values below 0.05 were considered significant. The likelihood ratio test in the univariate Cox regression model was used to test for differences and trend. The relapse-free and overall survival probabilities were calculated by the actuarial method of Kaplan and Meier (1958).

### RESULTS

### Analysis of *TP53* gene mutations in 222 breast tumours

Two-hundred and twenty-two female breast tumour specimens were studied. Altered migration patterns on SSCA, indicative of *TP53* gene mutations, were observed in 77 (35%) samples. The sequence data were successfully obtained on 214 tumours

© Cancer Research Campaign 1998

**Figure 1** Mutation spectrum of *TP53* gene alterations in 222 breast tumours. The four *silent* mutations [at codons 244, 256, 257 ($n$ = 2)] and the six cases with neutral *polymorphism* at codon 213 (exon 6) are shaded. Psr, protein stabilizing region; Zn, Zinc contact; DC, DNA contact. Exon 5, codon 126–186; exon 6, codon 187–224; exon 7, codon 225–261 and exon 8, codon 262–306

(see Table 1). Sixty-six tumour samples were successfully sequenced and 69 *TP53* gene mutations were found in these 66 tumour samples (see Table 1 and Figure 1). We observed 54 missense point mutations, comprising 78% of all mutations, and five mutations leading to a premature termination of the protein [one nonsense mutation (codon 196) and four deletions and/or insertions (codons 150, 216 and two times at codon 218, in exons 5 and 6)]. As expected, the majority (81%) of the mutations are transitions, and mutations of the amino acid arginine (Arg, $n$ = 20) are the most prevalent (34%). These mutations were distributed over 37 distinct codons and resided especially in exon 7 (41%). In addition, we observed six cases of polymorphism of codon 213 (exon 6) and four silent mutations (codons 244, 256 and two times codon 257; all within exon 7).

Forty-one out of 59 mutations (69%) were restrained to the conserved regions, and 25 mutations (42%) were within the zinc-binding domains (regions L2 and L3; Figure 1). Moreover, our analysis identified mutations in three of the seven amino acids important in direct DNA binding (i.e. codons 248, 273 and 280), in total 16 mutations were observed (Figure 1).

Recently, a LIA became available for the measurement of TP53 protein levels in cytosolic extracts. This assay, which detects both wild-type and mutant TP53 protein, is based on the principle that mutated TP53 has a prolonged half-life and is thus accumulated in the cell. In a subset of 151 primary breast tumour samples, the LIA values were related with the outcome of mutation analysis. The median TP53 level of 4.2 ng $mg^{-1}$ protein (range 0.0–176.0) in 46 tumours with missense mutations was higher (eightfold) than those levels measured in ten tumours with a silent mutation or in 91 tumours without a *TP53* gene alteration [median levels of 0.5 (range 0.0–11.9) or 0.4 (range 0.0–70.8 ng $mg^{-1}$ protein) respectively]. The level in tumours with deletions/insertions, however, was also low, i.e. 0.1 ng $mg^{-1}$ protein (range 0.0–0.16).

### *TP53* gene mutations related with tumour and patient characteristics and (relapse-free) survival of 177 patients

Of the 177 patients who were evaluable for analysis of relapse-free survival (RFS) and overall survival (OS), we observed 43 missense point mutations, four mutations leading to premature termination of the protein (one nonsense and three deletions and/or insertions) and six silent alterations in the primary tumours (Table 2). The frequency of *TP53* mutations in the primary tumours was not significantly related with tumour size or nodal status (Table 1) nor was there a relation with failure type (Table 2). The median RFS in the six patients with a mutation at codon 213 (neutral polymorphism) or a silent mutation (in exon 7) was shorter (median RFS = 11; range 7–107 months) than the RFS of the 47 patients with a mutation (median RFS = 34; range 3–160 months) and with the median RFS of 124 patients with wild-type *TP53* (median RFS = 48; range 2–183 months).

In an exploratory analysis the *TP53* gene mutations were stratified according to type of mutation, for example evolutionarily conserved regions, zinc-binding domains L2 (residues 163–195) and L3 (residues 236–251) of the protein, by residues that directly

© Cancer Research Campaign 1998

**Table 2** Patient and tumour characteristics and *TP53* gene mutations of patients with follow-up

| Number | Codon[a] | Change[b] Effect | Change[b] Site | Lia[c] (ng mg$^{-1}$ P) | Age (years) | T/N[d] | DFS[e] (months) | Site of metastasis[f] |
|---|---|---|---|---|---|---|---|---|
| *Missense* | | | | | | | | |
| 1 | 127 | Ser→Pro | | 2.0 | 49 | 1/1 | > 118 | |
| 2 | 135 | Cys→Arg | | 5.8 | 70 | 4/1 | 4 | SCCI |
| 3 | 138 | Ala→Val | | 4.2 | 67 | 2/12 | 8 | META |
| 4 | 145 | Leu→Arg | | n.d. | 61 | 3/0 | 42 | LRR |
| 5 | 151 | Pro→Ala | | 41.9 | 49 | 2/1 | 3 | META |
| 6 | 162 | Ile→Thr | | 0.2 | 64 | 1/1 | 122 | LRR |
| 7 | 163 | Tyr→Cys | L2 | 0.1 | 73 | 2/1 | 17 | SCCI |
| 8 | 173 | Val→Ala | L2 | 1.2 | 62 | 1/1 | > 100 | |
| 9 | 175 | Arg→His | L2/psr | 6.1 | 40 | 2/1 | 13 | META |
| 10 | 175 | Arg→His | L2/psr | 1.9 | 68 | 2/0 | > 72 | |
| 11 | 176 | Cys→Tyr | L2/Zn | 4.8 | 50 | 1/1 | 60 | META |
| 12 | 180 | Glu→Gly | L2 | 2.8 | 55 | 1/1 | 17 | META |
| 13 | 205 | Tyr→Phe | | 11.7 | 57 | 2/1 | 34 | SCCI |
| 14 | 212 | Phe→Leu | | n.d. | 42 | 1/0 | 36 | META |
| 15 | 218 | Val→Glu | | 13.0 | 75 | 2/2 | 34 | META |
| 16 | 220 | Tyr→Ser | | 4.1 | 54 | 2/0 | > 122 | |
| 17 | 232 | Ile→Ser | | 9.8 | 52 | 2/1 | > 116 | |
| 18 | 234 | Tyr→Cys | | 0.6 | 46 | 1/1 | > 54 | |
| 19 | 234 | Tyr→Cys | | 5.8 | 82 | 2/0 | > 108 | |
| 20 | 236 | Tyr→Cys | L3 | 0.1 | 72 | 2/x | 26 | META |
| 21 | 238 | Cys→Ser | L3/Zn | nd | 62 | 2/0 | 30 | META |
| 22 | 238 | Cys→Tyr | L3/Zn | 0.4 | 44 | 1/1 | 111 | LRR |
| 23 | 244 | Gly→Ser | L3 | 1.7 | 74 | 2/1 | 32 | META |
| 24 | 245 | Gly→Ser | L3/psr | 4.8 | 50 | 3/1 | 14 | META |
| 25 | 245 | Gly→Ser | L3/psr | 2.0 | 77 | 4/1 | 15 | META |
| 26 | 245 | Gly→Ser | L3/psr | n.d. | 47 | 3/1 | 18 | META |
| 27 | 246 | Met→Val | L3 | n.d. | 52 | 1/0 | > 113 | |
| 28 | 248 | Arg→Gln | L3/DNA | 176.0 | 72 | 3/1 | 4 | META |
| 29 | 248 | Arg→Gln | L3/DNA | 49.0 | 58 | 2/1 | 6 | META |
| 30 | 248 | Arg→Gln | L3/DNA | 26.3 | 70 | 3/0 | 12 | META |
| 31 | 248 | Arg→Gln | L3/DNA | 87.3 | 75 | 1/1 | 3 | D-BRCA |
| 32 | 248 | Arg→Trp | L3/DNA | n.d. | 51 | 2/0 | 46 | META |
| 33 | 248 | Arg→Trp | L3/DNA | 0.3 | 57 | 3/0 | > 141 | |
| 34 | 254 | Ile→Ser | | n.d. | 52 | 1/0 | > 160 | |
| 35 | 257 | Leu→Pro | | 1.1 | 61 | 1/1 | 50 | D-BRCA |
| 36 | 272 | Val→Met | | 9.0 | 34 | 2/1 | 10 | META |
| 37 | 273 | Arg→His | DNA | 20.6 | 57 | x/1 | 6 | META |
| 38 | 273 | Arg→His | DNA | 6.0 | 39 | 2/0 | 34 | META |
| 39 | 275 | Cys→Tyr | | 12.7 | 53 | 2.0 | > 100 | |
| 40 | 280 | Arg→Thr | DNA | 0.2 | 46 | 3/0 | 25 | META |
| 41 | 280 | Arg→Thr | DNA | 3.3 | 73 | 2/1 | 23 | D-BRCA |
| 42 | 282 | Arg→Gly | psr | 5.7 | 59 | 2/1 | 44 | META |
| 43 | 307 | Gly→Asp | | 0.3 | 54 | 2/0 | 7 | META |
| *Deletion/insertion/nonsense* | | | | | | | | |
| 44 | 150 | Frameshift; 449–467*, 19bp del | | 0.2 | 61 | 2/0 | 45 | META |
| 45 | 196 | Arg→stop | n | n.d. | 38 | 1/0 | 26 | MAM2 |
| 46 | 218 | Frameshift; 653–657, 5bp d/i 4bp | | 0.0 | 54 | 4/1 | 21 | META |
| 47 | 218 | Frameshift; 654 ins 5bp | | n.d. | 75 | 1/0 | 42 | D-BRCA |
| *Silent* | | | | | | | | |
| 48 | 213 | Arg→Arg | | 11.9 | 42 | 4/1 | 7 | SCCI |
| 49 | 213 | Arg→Arg | | 0.3 | 77 | 3/1 | 9 | META |
| 50 | 213 | Arg→Arg | | 0.7 | 44 | 1/1 | 11 | META |
| 51 | 213 | Arg→Arg | | 0.4 | 73 | 2/2 | 17 | META |
| 52 | 213 | Arg→Arg | | 0.7 | 68 | 2/0 | > 107 | |
| 53 | 244 | Gly→Gly | L3 | 0.0 | 53 | 2/0 | 11 | SCCI |

[a]Double mutants, number 8 has an additional frameshift at codon 216 (d/i) and number 25 has an additional mutation at codon 253 (Thr→Ala). [b]Site, L2/L3, loop 2 or loop 3; psr, protein stabilizing region; Zn, zinc-binding domain; DNA, direct contact with DNA. Del/d, deletion; ins/i, insertion; n, nonsense; *position of base. [c]LIA, luminometric immunoassay (see Patients and methods). [d]T tumour size (1, ≤ 2 cm; 2, 2–5 cm; 3/4, > 5 cm); N, nodal status (0, node negative; 1, node positive; x, unknown). [e]DFS, disease-free survival in months. [f]Site of relapse: SCCI, distant nodes; META, distant metastasis; LRR, local regional relapse; D-BRCA, dead without evidence of recurrent disease. Only patients with complete tumour characteristics available were included in this table.

**Table 3** Cox univariate (relapse-free) survival analysis of 177 breast cancer patients, stratified by TP53 mutation type

| | | Five-year RFS | | | Five-year OS | | |
|---|---|---|---|---|---|---|---|
| **Patient group** | ***n*** | **RHR** | **95% CL** | ***P*-value** | **RHR** | **95% CL** | ***P*-value** |
| WT | 124 | 1 | | | 1 | | |
| All mutations | 53 | 1.7 | 1.1–2.6 | 0.01 | 1.8 | 1.1–2.8 | 0.02 |
| WT | 124 | 1 | | | 1 | | |
| Non-conserved | 19 | 1.6 | 0.96–2.6 | n.s. | 1.8 | 0.95–3.6 | n.s. |
| Conserved region | 34 | 1.9 | 1.1–3.5 | 0.03 | 1.7 | 1.0–3.0 | 0.05 |
| WT | 124 | 1 | | | 1 | | |
| Outside loops | 33 | 1.6 | 0.95–2.6 | n.s. | 1.7 | 0.97–2.9 | n.s. |
| Inside loops, L2 and L3 | 20 | 2.0 | 1.1–3.6 | 0.02 | 1.9 | 1.0–3.8 | 0.05 |
| WT | 124 | 1 | | | 1 | | |
| Non-direct DNA | 43 | 1.6 | 1.0–2.5 | 0.05 | 1.5 | 0.93–2.6 | n.s. |
| Direct DNA contact | 10 | 2.7 | 1.2–5.8 | 0.02 | 3.4 | 1.5–7.6 | 0.002 |

WT, wild-type *TP53*. RHR, relative hazard rate. 95% CL, 95% confidence interval. For the explanation of codons involved in silent mutation, conserved regions, loops L2 and L3 and direct DNA contact see Figure 1 and Results.

contact DNA (Table 3) and by occurrence of silent mutations. Cox univariate analysis, at 5 years, showed that both the relapse-free and overall survival of patients with *TP53* mutations in either the conserved regions, within L2 and L3, or the codons that directly contact DNA is significantly worse (relative hazard rate (RHR) for RFS: 1.9, 2.0, 2.7 and 4.1 respectively; see Table 3) than those patients without mutations (WT, wild type), or with mutations in codons that are not conserved or are outside L2 and L3.

Actuarial relapse-free and overall survival curves stratified by *TP53* status revealed that the *TP53* gene mutation was significantly ($P = 0.02$) associated with an increased risk of relapse with a RHR of 1.6 [95% confidence interval (CI): 1.1–2.4], but not with the rate of death [$P = 0.07$; RHR = 1.5 (CI = 0.97–2.2), shown in Figure 2A and B]. When stratified as the function of type of *TP53* mutation, only mutations at residues that directly contact DNA retained significance (RFS: $P < 0.05$ and OS: $P < 0.02$, respectively, shown in Figure 2C and D).

In a multivariate Cox regression analysis, which included age, menopausal status, lymph node status, tumour size, steroid hormone receptor status and c-*MYC* amplification, *TP53* gene mutation independently predicts poor RFS and OS with relative hazard rates of 1.8 ($P < 0.01$) and 1.6 ($P = 0.04$) respectively.

### DISCUSSION

The present prevalence of *TP53* mutations is in the range of the overall rate of *TP53* mutations of 15–71% (mean 25%; examined in 1452 breast tumour samples worldwide, by SSCA of exon 5–8, and reviewed by Hartmann et al, 1997). Evaluation of only exons 5 through 8 may, however, underestimate the overall prevalence of *TP53* mutations by 10–20% (Bergh et al, 1995; Hartmann et al, 1995). The mutations observed in this study resided mainly (41%) in exon 7. This high incidence is in accordance with the study of Anderson et al (1993) who also described a predominance of mutations in exon 7. The mutational 'hotspots' described in breast cancer (Greenblatt et al, 1994), i.e. codons Arg-175, Arg-248 and Arg-273 accounted for 3%, 17% and 7%, respectively, in this study. This differs from the prevalence of 6%, 7%, and 7%, respectively, summarized in the database established by Cariello et al (1994) on *TP53* gene mutations ($n = 365$) in human primary breast tumours. Interestingly, Børresen et al (1995) also showed a relatively lower frequency (3%) of mutations in codon 175 and this could imply a lower prevalence of codon 175 mutations in European women. When stratifying the mutations according to the evolutionarily conserved regions or functional domains, we observed that 41 out of 59 mutations (69%) were restrained to the conserved regions, which is in concordance with the percentage of mutations (73%) in this region summarized by Cariello et al (1994). Bergh et al (1995), who studied mainly tumours from node-negative patients, observed a smaller number of mutations in these conserved regions, i.e. 30 out of 65 (46%). In our smaller number of node-negative patients 13 out of 21 mutations were in the conserved regions (62%). Twenty-five mutations (42%) in this study were within the structural regions L2 and L3, which is in accordance with data observed by Børresen et al (1995).

As expected, the median TP53 level of tumours with missense mutations was higher (eightfold) than levels of tumours with a silent mutation or without a gene alteration, but also with four tumours with deletions/insertions. This last result shows that low levels of TP53 measured by LIA, and probably also by immunohistochemistry, are not always indicative of a normal *TP53* gene status and the data should be interpreted with care.

An unexpected finding was that the median RFS in the six patients with a neutral polymorphism at codon 213 or a silent mutation was shorter (median RFS = 11 months) than the RFS of the 47 patients with a mutation (median RFS = 34 months) and with the median RFS of 124 patients with wild-type *TP53* (median RFS = 48 months). Shiao et al (1995) also reported that two patients with the A→G transition in codon 213 experienced a very poor survival, but more studies on this silent third base mutation in Arg-213 will be needed to clarify these observations. We also observed that the different *TP53* gene mutations in primary breast cancer could be related to differences in (relapse-free) survival, for example our study shows that mutations that directly contact DNA are related with a poor (relapse-free) survival of breast cancer patients. This does not agree with data from Børresen et al (1995). These authors reported that, in a multicentre study, patients with TP53 mutations in the zinc-binding domains (L2 and L3) had a poor prognosis. One possible explanation for the observed difference could be the shorter follow-up time in their study (median: 40 months vs 115 months in this study). In conclusion, the analysis of type and location of *TP53* alterations can be used to select residues

© Cancer Research Campaign 1998

**Figure 2** Actuarial relapse-free (**A** and **C**) and overall (**B** and **D**) survival as a function of *TP53* gene mutation. **A** and **B** WT, wild-type ($n$ = 124 patients); mut, mutated in exons 5 to 8 ($n$ = 53 patients). **C** and **D** non dir DNA, no direct contact of the affected codon with the DNA strand ($n$ = 43 and ten patients respectively).

that could be of prognostic value. If these data are confirmed by other investigators in larger studies, the use of artificially or naturally created restriction sites or allele-specific oligo techniques can facilitate the detection of these DNA contact residues.

### ACKNOWLEDGEMENTS

The authors wish to thank Henk Portengen, Doorlene van Tienoven and Drs Hans de Witte for excellent contribution to this project, and Drs Marion Meijer-van Gelder for clinical follow-up data. This work was supported through grants of the Dutch Cancer Society (DDHK 92-4/96–1234).

### REFERENCES

Andersen TI and Borresen AL (1995) Alterations of the TP53 gene as a potential prognostic marker in breast carcinomas. *Diagnos Mol Pathol* **4**: 203–211

Andersen TI, Holm R, Nesland JM, Heimdal KR, Ottestad L and Borresen AL (1993) Prognostic significance of TP53 alterations in breast carcinoma. *Br J Cancer* **68**: 540–548

Bergh J, Norberg T, Sjogren S, Lindgren A and Holmberg L (1995) Complete sequencing of the p53 gene provides prognostic information in breast cancer patients, particularly in relation to adjuvant systemic therapy and radiotherapy. *Nature Med* **1**: 1029–1034

Berns PMJJ, Klijn JGM, Staveren Van IL, Portengen H, Noordegraaf E and Foekens JA (1992) Prevalence of amplification of the oncogenes c-myc, HER2/neu, and int-2 in thousand human breast cancers: relationships with steroid receptors. *Eur J Cancer* **28**: 697–700

Berns EMJJ, Klijn JGM, Smid M, Van Staveren IL, Look MP, Van Putten WLJ and Foekens JA (1996) TP53 and MYC gene alterations independently predict poor prognosis in breast cancer patients. *Genes Chromos Cancer* **16**: 170–179

Borresen AL, Andersen TI, Eyfjord JE, Cornelis RS, Thorlacius S, Borg A, Johansson U, Theillet C, Scherneck S and Hartman S (1995) TP53 mutations and breast cancer prognosis: Particularly poor survival rates for cases with mutations in the zinc-binding domains. *Genes Chromos Cancer* **14**: 71–75

Carbone D, Chiba I and Mitsudomi T (1991) Polymorphism at codon 213 within the p53 gene. *Oncogene* **6**: 1691

Cariello NF, Cui L, Beroud C and Soussi T (1994) Database and software for the analysis of mutations in the human p53 gene. *Cancer Res* **54**: 4454–4460

Cho Y, Gorina S, Jeffery PD and Pavletich NP (1994) Crystal structure of a p53 tumor suppressor-DNA complex: understanding tumorigenesis. *Science* **265**: 346–355

De Witte HH, Foekens JA, Lennerstrand J, Smid M, Look MP, Klijn JGM, Benraad T and Berns EMJJ (1996) Prognostic significance of TP53 accumulation in human primary breast cancer: Comparison between a rapid quantitative immunoassay and SSCP analysis. *Int J Cancer* **69**: 125–130

Eortc Breast Cancer Cooperative Group (1973) Standard for the assessment of hormone receptors in human breast cancer. *Eur J Cancer* **9**: 379–381

Friend S (1994) p53: A glimpse at the puppet behind the shadow play. *Science* **265**: 334–335

Greenblatt MS, Bennet WP, Hollstein M and Harris CC (1994) Mutations in the p53 tumor suppressor gene: clues to cancer etiology and molecular pathogenesis. *Cancer Res* **54**: 4855–4878

Haffner R and Oren M (1995) Biochemical properties and biological effects of p53. *Genes Dev* **5**: 84–90

Harris CC (1996*a*) Structure and function of the p53 tumor suppressor gene: Clues for rational cancer therapeutic strategies. *J Natl Cancer Inst* **28**: 1442–1455

Harris CC (1996*b*) The 1995 Walter Hubert Lecture – molecular epidemiology of human cancer: insights from the mutational analysis of the p53 tumour-suppressor gene. *Br J Cancer* **73**: 261–269

Hartmann A, Blaszyk H, McGovern RM, Schroeder JJ, Cunningham J, De Vries EM, Kovach JS and Sommer SS (1995) p53 Gene mutations inside and outside of exons 5–6: the patterns differ in breast and other cancers. *Oncogene* **10**: 681–688

Hartmann A, Blaszyk H, Kovach JS and Sommer SS (1997) The molecular epidemiology of p53 gene mutations in human breast cancer. *Trends Genet* **13**: 27–33

© Cancer Research Campaign 1998

Kaplan EL and Meier P (1958) Nonparametric estimation from incomplete observation. *J Am Stat Assoc* **53**: 457–481

Kinzler KW and Vogelstein B (1996) Life (and death) in a malignant tumour. *Nature* **379**: 19–20

Levine AJ, Momand J, Finlay CA (1991) The p53 tumour suppressor gene. *Nature* **351**: 453–456

Prives C (1994) How loops, β sheets, and a helices help us to understand p53. *Cell* **78**: 543–546

Putten VWLJ, Klijn JGM, Meijer-Van Gelder ME, Look MP and Foekens JA (1996) Multiparameter analysis of prognostic factors in breast cancer. *Breast Cancer* 209–215

Shiao YH, Chen VW, Scheer D, Wu XC and Correa P (1995) Racial disparity in the association of p53 gene alterations with breast cancer survival. *Cancer Res* **55**: 1485–1490

Velculescu VE and El-Deiry WS (1996) Biological and clinical importance of the p53 tumor suppressor gene. *Clin Chem* **42**: 858–868

*© Cancer Research Campaign 1998*